# A Randomized Study Comparing the Efficacy of Three Hepatitis B Vaccine Induction Regimens in Adult Patients with Hematological Malignancies

**DOI:** 10.4274/tjh.2015.0079

**Published:** 2016-08-19

**Authors:** Zübeyde Nur Özkurt, Elif Suyanı, Rauf Haznedar, Münci Yağcı

**Affiliations:** 1 Gazi University Faculty of Medicine, Department of Hematology, Ankara, Turkey

**Keywords:** hepatitis B, Vaccine, Hematological malignancies

## Abstract

**Objective:**

Non-responsiveness to hepatitis B virus (HBV) vaccines is not rare in hemato-oncological patients due to disease-associated or treatment-induced immune suppression. Although different strategies have been employed to improve the response rates, to date there is not an approved schedule for HBV immunization in patients with hematological malignancies. We designed a prospective randomized study to evaluate the efficacy of 3 different induction regimens for HBV vaccination.

**Materials and Methods:**

In the standard-dose (SD) group, total vaccine dose delivered was 40 µg and patients were vaccinated with 20 µg at weeks 0 and 4. In the high-dose dose-intensive (HDDI) group, total vaccine dose delivered was 80 µg and patients were vaccinated with 40 µg at weeks 0 and 4. In the high-dose time-intensive (HDTI) group, total vaccine dose delivered was 80 µg and patients were vaccinated with 20 µg at weeks 0, 2, 4, and 6.

**Results:**

In a cohort of 114 patients, 38.6% responded to HBV vaccination. The response rate in the SD arm, HDDI arm, and HDTI arm was 26.2%, 29.7%, and 44.4%, respectively (p>0.05). Age was the only variable identified as having a negative impact on response.

**Conclusion:**

Short of achieving statistical significance, a higher response rate was observed in the HDTI arm. Therefore, this study supports a high-dose, time-intensive HBV vaccine induction regimen in patients with hematological malignancies who are not on chemotherapy.

## Abstract (tr)

**Amaç:**

Hematolojik maliniteli hastalarda hastalık veya tedavi ilişkili immün baskılanma yüzünden Hepatit B virüsü (HBV) aşısı yanıtsızlığı nadir değildir. Aşı yanıtını düzeltecek yöntemler denenmiş olsa da hematolojik maligniteli hastalarda kabul görmüş bir protokol henüz mevcut değildir.

**Gereç ve Yöntemler:**

Üç farklı HBV aşılama rejimini karşılaştıran ileriye dönük ve randomize bir çalışma tasarlandı. Standart doz (SD) grubuna toplam 40 µg olmak üzere 0. ve 4. haftada 20 µg HBV aşısı yapıldı. Yüksek doz-doz yoğun (YDDY) gruba toplam 80 µg HBV aşısını 0. ve 4. haftada 40 µg uygulandı. Yüksek doz-sık uygulama (YDSU) grubuna toplam 80 µg HBV aşısı 0., 2., 4. ve 6. haftalarda 20 µg yapıldı.

**Bulgular:**

Yüz on dört hastayı içeren bu çalışmada HBV aşısına yanıt %38,6 bulundu. Yanıt oranı SD, YDDY ve YDSU kolu için yanıt sırası ile %26,2, %29,7 ve %44,7 bulundu (p>0,05). Aşı yanıtı üzerine olumsuz etkili tek değişkenin yaş olduğu saptandı.

**Sonuç:**

İstatistiksel anlamı olmasa da YDSU kolunda HBV aşı yanıtı oransal olarak daha yüksek bulundu. Bu çalışmada elde edilen sonuçlar hematolojik maligniteli hastalarda yüksek doz ve daha sık uygulanan HBV aşılama rejiminin daha etkin olduğunu düşündürmektedir.

## INTRODUCTION

Hepatitis B virus (HBV) infection, the most common chronic viral infection in the world, can have serious clinical complications ranging from fulminant hepatitis to cirrhosis and hepatocellular carcinoma [1]. Vaccination is most effective in preventing HBV infection and complications. The complete vaccine series induce protective antibody levels in more than 95% of infants, children, and young adults. Protection has been estimated to last at least 20 years and is possibly lifelong [2]. It is generally held that patients with hematological malignancies are immunosuppressed, either as a result of the underlying hematological malignancy or due to treatment with chemotherapy. Immunosuppressive diseases like hematological malignancies are a risk factor for non-responsiveness to HBV vaccination. Despite low response rates, it is recommended that HBV-naive patients with hematological malignancies be immunized against HBV [3].

Although a rapid and effective strategy for HBV immunization of patients with hematological malignancies is highly desirable, to date there is not an approved schedule for these patients. Different strategies have been employed to improve the response rates in immunologically compromised patients, including HIV-infected adult patients, and in patients with chronic kidney disease [4,5,6]. We designed a prospective randomized study to evaluate the efficacy of 3 different induction regimens for HBV immunization in patients with hematological malignancies. In this study we aim to compare the results of the 3 different induction regimens for HBV vaccine at week 8. The basis of this deviation of omitting the consolidation dose at month 6, the current standard of care for routine HBV vaccination, was to decrease the drop-out rate due to stem cell transplantation, progression, relapse, death, frequent infections, and the effect of infection, antibiotics, and intravenous immunoglobulin on vaccination, and to allow more patients to be recruited into the study.

## MATERIALS AND METHODS

Patients with hematological malignancies followed by the Division of Hematology at Gazi University in Turkey between January 2008 and December 2013 were included in the study. Inclusion criteria were:

a. Age >18 years

b. Eastern Cooperative Oncology Group performance status ≥2,

c. Negative serology for HBsAg, anti-HBc, and anti-HBs,

d. Negative serology for hepatitis C and HIV,

e. Chemotherapy-naive patients,

f. Patients who achieved remission or stable disease after chemotherapy,

g. In subjects with a history of treatment, a time interval of at least 3 months from the last dose of chemotherapy and/or radiotherapy.

Exclusion criteria were:

a. History of prior HBV vaccination, 

b. Evidence of ongoing systemic infection,

c. Ongoing immunosuppressive therapy,

d. History of prior stem cell transplantation,

e. History of a non-hematological malignancy, except adequately treated squamous or basal cell carcinoma of the skin or cervical carcinoma in situ.

Chemotherapy regimens were categorized into 4 groups, namely alkylating agent-based, purine analog-based, chemo immunotherapy, and acute leukemia type, in an attempt to evaluate the effect of various drugs with different modes of action on vaccine response. Disease status at the time of vaccination was evaluated as untreated, in complete remission, or stable disease.

Before vaccination blood was drawn for complete blood count and IgG, IgA, and IgM levels. Absolute CD4, CD8, CD3+ CD56+, and CD4+ CD25+ counts were determined by flow cytometry (Becton Dickinson, FACSCalibur). Anti-HBs levels were planned to be measured at week 8. Patients who completed the vaccine schedule and had an anti-HBs serology at week 8 were considered eligible for response evaluation.

### Study Protocol

All patients participating in the study received a recombinant HBV vaccine (Genhevac B, Sanofi Pasteur) in the deltoid region, intramuscularly. Patients were randomized (1:1:1 ratio) into 1 of the 3 groups below to receive the hepatitis B vaccine. Groups were named based on the total vaccine dose delivered and vaccination frequency.

### Group 1: Standard dose (SD):

Patients were vaccinated with 20 µg at weeks 0 and 4; total vaccine dose was 40 µg.

### Group 2: High-dose dose-intensive (HDDI):

Patients were vaccinated with 40 µg at weeks 0 and 4; total vaccine dose was 80 µg.

### Group 3: High-dose time-intensive (HDTI):

Patients were vaccinated with 20 µg at weeks 0, 2, 4, and 6; total vaccine dose was 80 µg.

Patients who achieved anti-HBs levels of >10 IU/L were defined as vaccine responders. The primary endpoint was seroprotection rate at week 8 and the secondary endpoint was comparison of antibody levels in 3 different HBV vaccination regimens. The study protocol was approved by the Local Ethics Committee of Gazi University Faculty of Medicine and written informed consent was obtained from all subjects prior to study entry.

### Statistical Analysis

Statistical evaluation was done with SPSS 15. Data were described as numbers and percentages or medians and minimum-maximum, as appropriate. Chi-square test was used for evaluating categorical values, and Kruskal-Wallis and one-way ANOVA tests were used for continuous values in patient groups. All p-values were 2-sided with statistical significance at 0.05 alpha levels. Logistic regression analysis was used for multivariate analysis to evaluate the factors affecting the vaccination response.

## RESULTS

A total of 124 patients were randomized during the study period. Ten patients were excluded from the study due to cerebrovascular event before the first dose of the vaccine (1 patient) or not fulfilling the criteria for response evaluation after vaccination (9 patients). Characteristics of the patients are summarized in Tables 1 and 2. Significant differences existed only in CD4/CD8 ratio between the 3 vaccine groups.

### Response Evaluation at Week 8

**Figure 1:**
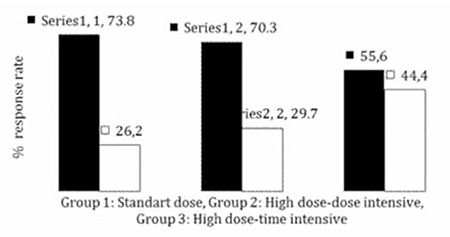
Response rates to 3 different vaccination regimens at week 8.

**Figure 2:**
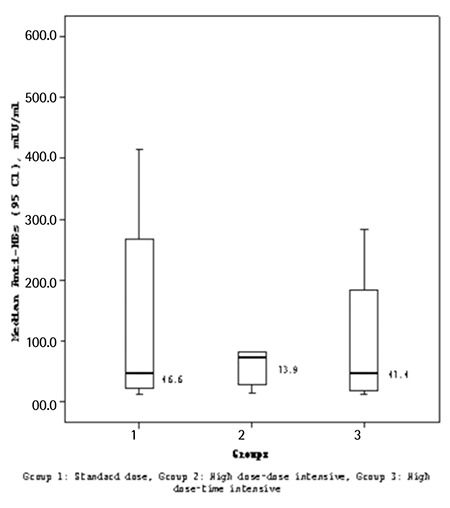
Median antibody concentration response to 3 different regimens at week 8.

At the end of the study, 114 patients were eligible for response evaluation. Overall, 44 of 114 patients responded, with a response rate of 38.6% at week 8. On the other hand, 61.4% of the patients did not respond to HBV vaccination. The response rate in the SD arm, HDDI arm, and HDTI arm was 26.2%, 29.7%, and 44.4%, respectively (Figure 1). The high response rate in the HDTI arm did not translate to statistical significance, possibly due to relatively low patient numbers (p>0.05). The median antibody concentration (MAC) in the SD arm, HDDI arm, and HDTI arm was 46.6 IU/L (12.4-706), 73.95 IU/L (14.7-779), and 47.4 IU/L (11.6-779), respectively (Figure 2). The differences in MAC between groups were not statistically significant. Among the variables evaluated, only age had a negative impact on response (p<0.001). Other clinical variables such as sex, disease type, disease status, treatment status, type of chemotherapy, radiotherapy, time from diagnosis to vaccination, and time from last treatment to vaccination had no impact on antibody response. Similarly IgG, IgM, and IgA levels and absolute CD4, CD8, CD3+ CD56+, and CD4+ CD25+ numbers did not influence the response. No serious adverse effects attributable to vaccination were identified.

## DISCUSSION

In this study, overall response rate, defined as anti-HBs of >10 IU/L at week 8, was 38.6%. The response rate in the SD arm, HDDI arm, and HDTI arm was 26.2%, 29.7%, and 44.4%, respectively. Short of achieving statistical significance, a higher response rate was observed in the HDTI arm. The MAC in the SD arm, HDDI arm, and HDTI arm was not statistically significant. Among the variables evaluated, only age had a negative impact on response.

HBV reactivation is a common complication in HBsAg-positive patients undergoing immunosuppressive anticancer therapy. The clinical consequences of HBV reactivation are observed as asymptomatic liver function disturbances, liver failure, and delay or premature cessation of chemotherapy courses with adverse prognostic consequences for the hematological disease. It is strongly recommended that all hemato-oncological patients be screened for HBV markers and immunization against hepatitis B be performed in HBV-naive patients when appropriate [3,7].

Conducting HBV vaccination trials in adult patients with hematological malignancies is troublesome. Heterogeneity of both the underlying hematological conditions and chemotherapy regimens, maintenance therapies, relapse of the disease, and salvage regimens including high-dose chemotherapy with stem cell support make the situation more complex. Therefore, recruiting a sufficient number of patients for randomized trials and multivariate analysis requires the active collaboration of centers, especially in developing countries.

As a result of these difficulties, the number of HBV vaccination trials in patients with hematological malignancies is very limited and mostly confined to pediatric patients with acute leukemia [8,9,10,11]. The data supporting HBV vaccination almost completely come from general vaccination strategies and no evidence-based recommendations for the dose, frequency, and timing of HBV vaccination in adult hematological patients are available.

The risk of HBV transmission, just after the diagnosis or during the chemotherapy of patients, is high due to frequent transfusions and interventions. Immediate vaccination is highly desirable; however, disease and chemotherapy may compromise antibody response. If the vaccination is postponed until after the chemotherapy, disease and chemotherapy-related immunosuppression are lessened and probability of response increases, but active protection from HBV during the high-risk period is missed.

Accelerated vaccination strategies could be useful for at-risk groups in terms of rapid seroconversion and increasing adherence. Studies conducted in high-risk healthy adults, drug users, lung transplantation candidates, and HIV-infected patients elicited similar or better anti-HBs responses and could be advantageous for the short term in this population. However, additional studies on long-term protection and effectiveness of accelerated schedules are necessary [12,13,14,15].

Non-responsiveness to HBV vaccine is not rare in hemato-oncological patients due to disease-associated or treatment-induced immune suppression. There are a number of means to augment the immune response to HBV immunization in non-responders, including adding additional doses, doubling the vaccine dose, intradermal injection of the vaccine, combination with granulocyte-macrophage colony-stimulating factor (GM-CSF), and use of new, more immunogenic HBV vaccines [16]. We have identified 2 studies on improving serological response to HBV vaccine in adult patients with hematological malignancies. First, Pullukcu et al. carried out a non-randomized study in 42 HBV-naive hematology patients during chemotherapy. Patients were administered a 20 µg HBV vaccine on days 0, 14, and 28. Overall, 23.8% of the patients responded to this accelerated schedule during chemotherapy [17].

The second study was a randomized one comparing the efficacy of a single dose of 40 µg HBV vaccine with one course of 40 µg HBV vaccine after 5 µg/kg recombinant GM-CSF injection in 94 patients with lymphoproliferative disorders (LPDs). Although the seroprotection rate was higher in the GM-CSF + HBV vaccine group (25.5% vs. 17%), the difference did not reach a significant level. In multivariate analysis, age was the only predictor of achieving a seroprotective response. The authors concluded that in LPDs, the response to HBV vaccine is impaired and GM-CSF may enhance the response rate to HBV vaccine [18].

The available data are far from allowing definite recommendations. However, some useful information about the dose, frequency, and timing of the vaccination may be collected for the design of future studies in adult patients with hematological disease. First of all, response to standard HBV vaccination is impaired and doubling the vaccine dose per injection does not increase the response rate at week 8. On the other hand, frequent antigenic stimulation seems to induce better immune response than a standard schedule. Thus, an accelerated vaccination schedule is feasible in this patient group.

The durability of the response, even in stable disease conditions, is another factor that needs to be taken into consideration. In our previous study, some of the patients lost their seroprotective anti-HBs responses beginning at the sixth month of the vaccination [18]. Therefore, we suggest that a single booster dose of vaccine should be given to responders at the sixth month of vaccination. Finally, response rate to HBV vaccination during chemotherapy is low even though a frequent injection scheme is used [17]. Moreover, anti-HBs response may be lost during a chemotherapy course [19]. Whether patients who lost anti-HBs response during chemotherapy need re-vaccination or a single booster dose of vaccine to reinduce antibody production is not known.

The findings from this study suggest a time-intensive approach, at 20 µg biweekly of 3-4 doses of HBV vaccine, for the design of future studies of adult patients with hematological disease who are not on chemotherapy.

## Ethics

Ethics Committee Approval: The study protocol was approved by the Local Ethics Committee of Gazi University Faculty of Medicine; Informed Consent: Written informed consent was obtained from all subjects prior to study entry.

## Figures and Tables

**Table 1:**
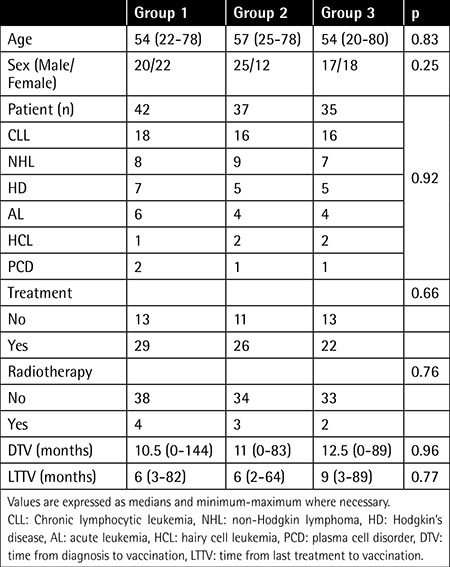
Baseline demographic characteristics of the study patients.

**Table 2:**
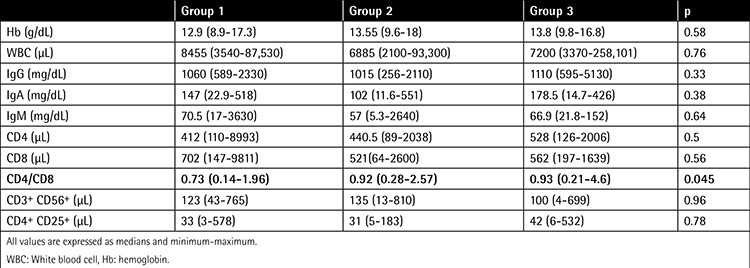
Baseline laboratory characteristics.
